# Predicting Falls in Long-term Care Facilities: Machine Learning Study

**DOI:** 10.2196/35373

**Published:** 2022-04-01

**Authors:** Rahul Thapa, Anurag Garikipati, Sepideh Shokouhi, Myrna Hurtado, Gina Barnes, Jana Hoffman, Jacob Calvert, Lynne Katzmann, Qingqing Mao, Ritankar Das

**Affiliations:** 1 Dascena Inc. Houston, TX United States; 2 Juniper Communities Bloomfield, NJ United States

**Keywords:** vital signs, machine learning, blood pressure, skilled nursing facilities, independent living facilities, assisted living facilities, fall prediction, elderly care, elderly population, older adult, aging

## Abstract

**Background:**

Short-term fall prediction models that use electronic health records (EHRs) may enable the implementation of dynamic care practices that specifically address changes in individualized fall risk within senior care facilities.

**Objective:**

The aim of this study is to implement machine learning (ML) algorithms that use EHR data to predict a 3-month fall risk in residents from a variety of senior care facilities providing different levels of care.

**Methods:**

This retrospective study obtained EHR data (2007-2021) from Juniper Communities’ proprietary database of 2785 individuals primarily residing in skilled nursing facilities, independent living facilities, and assisted living facilities across the United States. We assessed the performance of 3 ML-based fall prediction models and the Juniper Communities’ fall risk assessment. Additional analyses were conducted to examine how changes in the input features, training data sets, and prediction windows affected the performance of these models.

**Results:**

The Extreme Gradient Boosting model exhibited the highest performance, with an area under the receiver operating characteristic curve of 0.846 (95% CI 0.794-0.894), specificity of 0.848, diagnostic odds ratio of 13.40, and sensitivity of 0.706, while achieving the best trade-off in balancing true positive and negative rates. The number of active medications was the most significant feature associated with fall risk, followed by a resident’s number of active diseases and several variables associated with vital signs, including diastolic blood pressure and changes in weight and respiratory rates. The combination of vital signs with traditional risk factors as input features achieved higher prediction accuracy than using either group of features alone.

**Conclusions:**

This study shows that the Extreme Gradient Boosting technique can use a large number of features from EHR data to make short-term fall predictions with a better performance than that of conventional fall risk assessments and other ML models. The integration of routinely collected EHR data, particularly vital signs, into fall prediction models may generate more accurate fall risk surveillance than models without vital signs. Our data support the use of ML models for dynamic, cost-effective, and automated fall predictions in different types of senior care facilities.

## Introduction

### Background

Falls are a serious and complex safety concern, leading to mortality, morbidity, and increased health care costs associated with aging. Accidents are the fifth leading cause of death in older adults, and falls account for two-thirds of all accidental deaths [1]. Individuals who live in institutions fall more often (1.5 falls per bed per year) than community-living individuals, of whom the latter are generally healthy older people [1]. Between these 2 groups, it is estimated that 60% will experience a fall each year [2]. Most falls have a multifactorial origin. Previously reported fall risk factors include gait impairment, balance impairment, age, sex, cognitive decline, diminished vision, fall history, medications that affect the central nervous system, and several comorbidities [3-10]. Current fall risk profiles in nursing homes rely primarily on strength, gait, and balance measures [11]. Frequent administration and quantification of instruments that consider comprehensive risk factors create a challenge both in terms of impeding workflow and interpreting results. Evidence for the best choice for fall risk assessment in long-term facilities remains limited [2,12,13].

Electronic health records (EHRs) contain routinely collected real-time information that represents most fall risk factors and thus offer the potential for dynamic surveillance of senior residents in long-term facilities to identify short-term fall triggers. Although the wide range of fall risk factors embedded in EHR data poses methodological challenges to most traditional statistical approaches, machine learning algorithms (MLAs) can screen a multitude of interacting risk factors from big data. Machine learning (ML) is a subfield of artificial intelligence that can use sample data to build a model for predicting future outcomes or identifying hidden patterns of intrinsic structures within input data without explicit programming or data engineering. The two most commonly used ML methods are supervised and unsupervised learning. Supervised learning trains algorithms based on labeled training data, whereas the unsupervised learning approach does not require labeled training and can find structures within the data. Several EHR-based MLAs have been developed for fall risk predictions in hospitalized patients [14-18]. Few studies have explored the utility of ML approaches for senior residents in community-dwelling or long-term assisted living facilities [19-23]. Here, we developed an EHR-based supervised ML model using a gradient boosting (Extreme Gradient Boosting [XGBoosting]) algorithm to evaluate fall incidents within a 3-month window. By implementing advanced MLAs on EHR data from different types of long-term care facilities, we expected that our model would uncover the impact of a wide range of clinical and pathophysiological fall predictors across heterogeneous cohorts. We also hypothesize that these MLAs will outperform traditional fall risk assessments and standard ML techniques that are less compatible with EHR data in terms of dealing with missing data and class imbalances. Our previous studies with EHR data have shown that XGBoosting outperforms other ML models, such as logistic regression and simple forms of neural network-based models [24].

As most residents at long-term care facilities are at heightened risk of falls, more accurate short-term risk predictions would help identify individuals who may require more assistance with daily activities and enable care practices that are tailored to address short-term changes in fall risk and provide more dynamic fall risk profiles of residents for staff. Although previous research has primarily focused on identifying factors that increase the risk of falls, special emphasis must be placed on identifying factors that can reduce fall risk. In this context, it is critical to explore both the positive and negative associations between individual predictors and fall risk.

### Objective

The primary objective of this study is to determine the utility of ML in predicting short-term falls in long-term senior care settings and determine whether performance accuracy remained consistent in different types of facilities that are characterized by different levels of residents’ frailty and staff care (independent living, assisted living, and nursing homes). The inclusion of various measurements associated with vital signs, in addition to traditional risk factors that are incorporated into standard fall risk assessments, was one of the key designs of our ML models. Vital sign measures, such as blood pressure and respiratory rate, are dynamic parameters that reflect real-time changes in physiological function because of aging, frailty, different diseases, and treatments [25]. Although changes in vital signs are recognized as potential precursors to falls [26], the predictive value of these variables for fall risk in long-term senior care facilities has not been fully explored.

## Methods

### Data Source and Inclusion and Exclusion Criteria

**Figure 1:**
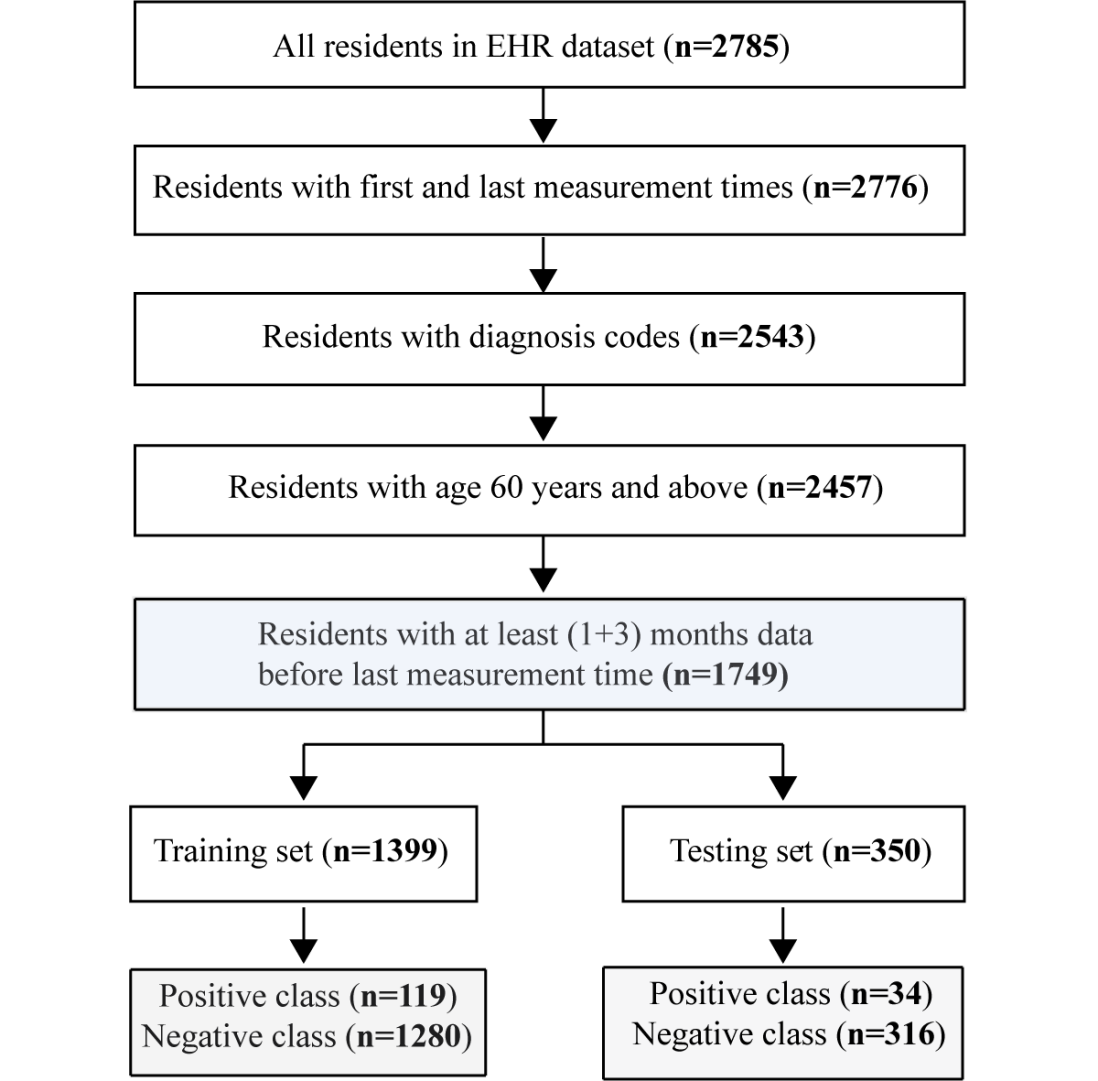
Participant encounter inclusion and exclusion diagram. EHR: electronic health record.

This study used data collected from a proprietary database containing EHR data from senior living communities (Juniper Communities, LLC) in the United States. The Juniper facilities included in this study were skilled nursing facilities, independent living facilities, assisted living facilities, and other non-major facilities without specific designations. Data were extracted from 2007 to 2021. Data were deidentified in compliance with the Health Insurance Portability and Accountability Act. As this study constituted nonhuman participants research per 45 Code of Federal Regulations 46.102, institutional review board approval was not required. Initially, the Juniper EHR contained data from 2785 residents. The first step of the filtration process removed residents who did not have the first measurement time, the last measurement time, or any EHR data, including diagnostic codes. We then excluded all residents age <60 years. Finally, we removed all residents who did not have at least 1 month of data available before the MLA runtime, defined as the time at which our algorithm predicted a fall. Figure 1 shows the participant inclusion and exclusion diagram.

**Figure 2:**
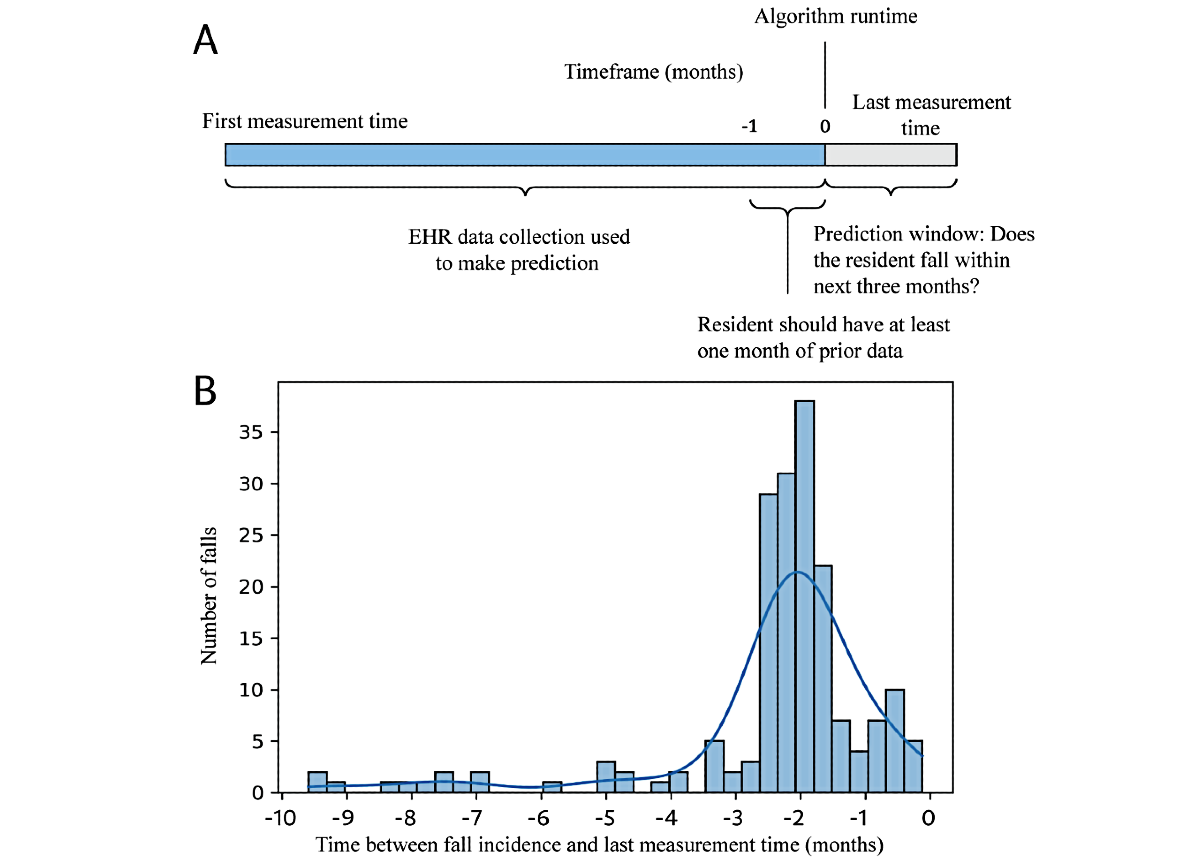
(A) Study design timeline. (B) Selection of the optimal prediction window based on the distribution of the fall incidence time. EHR: electronic health record.

Participants and fall incidence (positive cases) were identified according to both the International Classification of Diseases (ICD), Ninth Revision, and the ICD, Tenth Revision, as the EHR included resident data from October 2015. These codes included W00-W119 and R29.6 [27]. For a portion of the study cohort that did not have fall ICD codes, fall incidences were identified from string searching progress notes with fall-related strings, such as “fall” and “on the floor.” To meet the gold standard definition of our study, the fall had to occur within a 3-month period before the last measurement, as shown in (Figure 2A). The last measurement time was defined as the time at which data were collected from the resident. We used the distribution of the time differences between the fall incidents and the last measurement time (Figure 2B) of our cohort as a guideline for selecting the prediction window. We determined that the selection of a 3-month prediction window offered a good trade-off between maximizing the number of positive cases; that is, participants who experienced a fall within the given time while remaining within a reasonably short prediction window. A shorter prediction window reduces the number of positive cases, leading to a more imbalanced data set.

### ML Input Features for Fall Prediction

We conducted a literature search to gather previously reported fall risk factors and determine whether they could be identified within the EHR system based on relevant ICD codes, string searches, or keyword queries. We included several major risk factors such as age, sex, previous fall history, weakness, dizziness, cognitive impairment, dementia, depression, impaired mobility, and gait or balance abnormalities [3,28,29]. The fall history was included as the time difference between the MLA runtime and the most recent history of falls normalized to a year. In addition to dementia, depression, and mood disorders, we included other comorbidities [30] and medications implicated in fall risk [31-33] (Table S1 in Multimedia Appendix 1). Medications included benzodiazepines [9,34], antiepileptics [35], angiotensin-converting enzyme inhibitors [32], antidepressants [9], antipsychotics [36], narcotics [37], diuretics [36,38], β-blockers [39], antihistamines [33], neuromuscular blocking agents [40], calcium channel blockers [32], antiarrhythmics [41], sedatives, and hypnotics [9]. The participants’ vital sign measures and laboratory results were queried using the key names in the EHRs. The complete list of features and associated ICD codes can be found in Tables S1 and S2 in Multimedia Appendix 1. The feature importance metric was used to preselect the best features for the ML models, reducing the number of features from 250 to 68. Bolded features in Table S1 in Multimedia Appendix 1 are those that were preselected on the feature importance metric. These features included age, sex, specific vital sign measurements (diastolic and systolic blood pressure, heart rate, respiratory rate, and temperature), specific physical and movement features (height, weight, history of falls, and lower extremity fracture or dislocation), specific comorbidities (hypertension, chronic heart failure, stroke and cerebrovascular, and number of active diseases), and medications (benzodiazepines, angiotensin-converting enzyme inhibitors, antiepileptic and anticonvulsants, and total number of active medications). In addition to feature importance, the Shapely Additive Explanations (SHAP) analysis enhances the interpretability of the model by showing positive and negative associations and their strengths between individual features and fall risk.

For vital sign measurements, 4 months of data were used before the algorithm runtime. We filtered out data (vital signs and laboratory measures) that were identified as extreme outliers using the physiological minimum and maximum values. After removing these outliers, summary statistics, including minimum (min), maximum (max), mean, standard deviation (SD), last measurement (last), and the number of measurements (number), were used as input features. We calculated the summary statistics of the patient data over the last 1 month. Features related to comorbidities or medications were added as either previous or current comorbidities and medications. Given that our data did not provide structured information about medication dosage, we were not able to include dosage as an input feature.

### Comparison With Standard of Care

An internal fall risk assessment conducted by Juniper Communities staff served as a comparator. Participant scores (0-25) were tallied from several items, including the level of consciousness or mental status, history of fall, ambulation elimination, vision, gait, balance, medications, systolic blood pressure, and previous predisposition (eg, vertigo, obesity, osteoporosis, or Parkinson disease).

### ML Model

Our primary model, XGBoosting, was a gradient boosting algorithm [42] implemented in Python [43]. XGBoosting combines the results from various decision trees to obtain the prediction scores. Within each decision tree, the resident population was split into successively smaller groups, as each tree branch divided the residents who entered it into 1 of 2 groups based on their covariate value and a predetermined threshold. Fall residents were represented at the end of the decision tree, which were a set of leaf nodes. After the XGBoosting model was trained, successive trees were developed to improve the accuracy of the model. Successive iterations of trees use gradient descent on the prior trees to minimize the error of the next tree that was formed. XGBoosting has been shown to exhibit excellent performance for a wide range of classification problems in acute and chronic conditions [44-48]. For comparison with the structurally complex XGBoosting model, logistic regression and multilayered perceptron models were also trained and tested. A multilayered perceptron is a common network architecture with feed-forward neural networks composed of several layers of nodes with unidirectional connections. Unlike the XGBoosting model, logistic regression and multilayered perceptron models are unable to incorporate missing data; therefore, the median of observation was used for imputation of the features. In addition, we standardized our data for both the logistic regression and multilayered perceptron models. All the 3 models were trained using the same 68 inputs. The development environment of our MLAs (software package, library, and version) is summarized in Table S3 in Multimedia Appendix 1.

We used a standard approach to train ML models. We partitioned the data set into a train:test ratio of 80:20 with stratified sampling because the positive class was relatively small with respect to the negative class. Both the training and test sets included a random mix of all four types (skilled nursing facilities, assisted living facilities, independent living facilities, and others) of long-term facilities within Juniper Communities. All the models underwent hyperparameter selection using a 5-fold cross-validation grid search. The optimization of hyperparameters was confirmed by evaluating the area under the receiver operating characteristic (AUROC) curve for different combinations of hyperparameters included in the grid search. For XGBoosting, the optimization parameters were the maximum tree depth, regularization term (lambda), scale positive weight, learning rate, and number of estimators. The scale positive rate can be readily optimized within the XGBoosting algorithm to handle class imbalance in the data set (a lower number of residents who experienced a fall). We used a parameter space of (4, 6, 8, 10) for tree depth, (0.5, 1.0, 2, 3) for lambda, (1, 3, 5, 7, 9, 11, 13) for scale positive weight, (0.0001, 0.001, 0.01, 0.1) for learning rate, and (50, 75, 100, 125, 150, 500) for the number of estimators. The XGBoosting model used 6, 1.0, 13, 0.001, and 75 as the optimized values for the aforementioned parameters. For logistic regression, the optimization parameters were the penalty term, class weight, optimization problem solver, and inverse of regularization strength. The optimization parameters of the multilayered perceptron model included the maximum iteration, hidden layer size, and learning rate. For the logistic regression and multilayered perceptron models, missing values were handled using various imputation approaches. Missing measures of vital signs were imputed using the forward and backward filling approaches. For all other features, the mean measurement of the features across all the training set data was used for the imputation. For the logistic regression model, the inputs were scaled using a standard scaler from scikit-learn. The optimization algorithms for the logistic regression model included the limited-memory Broyden-Fletcher-Goldfarb-Shanno algorithm and L2 regularization. The multilayered perceptron model incorporated a hidden layer of size 250. The convergence of the solver iteration was determined either by reaching a maximum number (100) of iterations or by reaching a value of 1e-9 for the tolerance optimization parameter. All other parameters were kept at default values from the scikit-learn multilayered perceptron classifier. The performance of each model was assessed against the test data set with respect to the receiver operating characteristic (ROC) curve, sensitivity, and specificity. The confidence intervals (CIs) for these metrics were constructed using 1000 bootstrapped samples. SHAP [49] analysis was performed to evaluate the feature importance.

### Exploratory Analyses

Several exploratory analyses were conducted in this study. In the first experiment, we examined how the performance of the XGBoosting, logistic regression, multilayered perceptron, and comparator changed after reducing the prediction window to 2 months. We conducted a secondary experiment in which we separated the training and testing sets based on the type of facility (skilled nursing, independent living, and assisted living facilities). In the first case, data from the skilled nursing facility were used as the testing set, whereas data from all other facilities were used for model training. In the second case, assisted living facility data were used as the testing set, whereas data from all other facilities were used for model training. Owing to the small number of positive cases, independent living facilities were not tested separately. We conducted a third experiment in which we modified the input features of all 3 ML models to evaluate their impact on the model performance. First, we removed vital signs from the input features. Then, we included only the vital signs and demographic information (age and sex) and removed all other features, such as fall history, comorbidities, and medical conditions.

## Results

### Data Set Characteristics

In total, 2785 residents were included in this study, of whom 153 (153/2785, 5.49%) fell within the 3-month prediction window of our algorithm, as defined by our gold standard. The number of women was approximately twice that of men. Group differences were calculated using an exact binomial test for noncontinuous variables and 2-tailed Welch *t* test for continuous variables to handle the unequal variance associated with the 2 groups. The fall incidents varied among the types of facilities (Table S4 in Multimedia Appendix 1). Skilled nursing facilities had the highest (49/489, 10%) and independent living facilities had the lowest (5/69, 7%) fall incidents. Table S5 in Multimedia Appendix 1 summarizes the demographic and diagnostic information for nonfall residents (negative cases; age: mean 85.7, SD 9.5 years) and fall residents (positive cases; age: mean 86.6, SD 8.2 years).

### Model Performance

**Figure 3:**
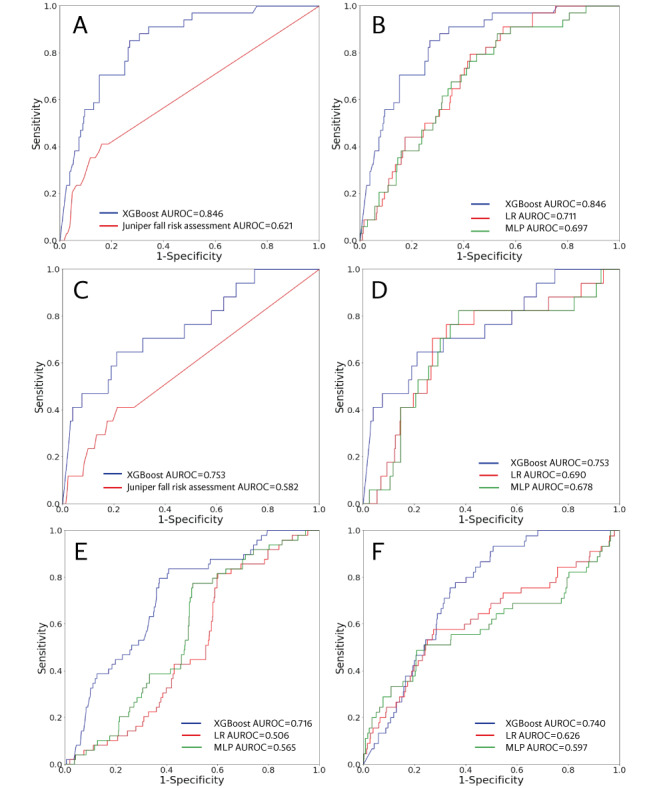
Row 1: Receiver operating characteristic (ROC) curves of the Extreme Gradient Boosting (XGBoost) model for three-month prediction compared with (A) the Juniper fall risk assessment and (B) other machine learning ML models. Row 2: ROC curves of the XGBoost model for a two-month prediction window compared with (C) the Juniper fall risk assessment and (D) other ML models. Row 3: ROC curves across different facilities. (E) Skilled nursing facility separated as a testing set and (F) Assisted living facility separated as a testing set. AUROC: area under the receiver operating characteristic; ML: machine learning; MLP: multilayered perceptron; LR: logistic regression.

**Figure 4:**
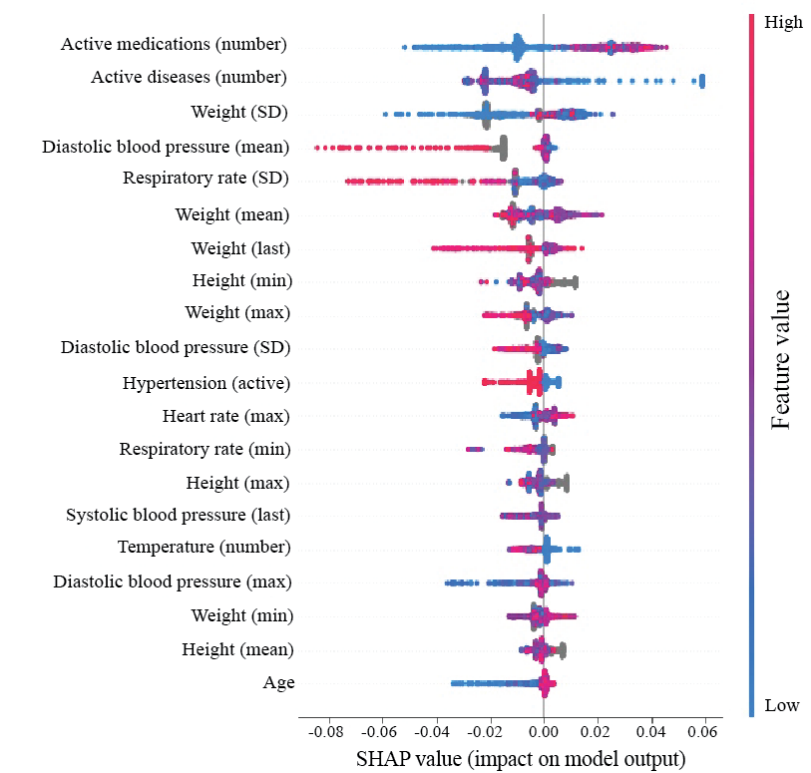
Feature correlations and distribution of feature importance for the Extreme Gradient Boosting (XGBoost) model at the three-month prediction window. The y-axis on the SHAP plot presents the features in order of importance from top to bottom. The SHAP values on the x-axis quantify the magnitude and direction in which each feature impacts the model prediction. SHAP: Shapely Additive Explanations.

The complete list of performance metrics for the MLAs and comparator is shown in Table 1. The ROC curves for the hold out test set are shown in Figures 3A and 3B. The XGBoosting model exhibited the highest performance with an AUROC of 0.846 for the prediction of falls within the next 3 months. The logistic regression model and the multilayered perceptron model demonstrated AUROCs of 0.711 and 0.697, respectively. The comparator (Juniper fall assessment) had an AUROC of 0.621. We selected an operating sensitivity of 0.70 for all 3 ML models and 0.35 for the comparator (based on the Juniper fall risk score threshold). The feature importance plot (Figure 4) shows the most important XGBoosting features, including the number of active medications, number of active diseases, SD of weight, mean diastolic blood pressure, and SD of respiratory rate. Younger age, lower weight fluctuations, and a larger number of active diseases were associated with a lower fall risk. A higher number of active medications was associated with a higher risk of falls. A higher mean value of diastolic arterial blood pressure (DiasAB) and higher fluctuations in respiratory rate were associated with lower fall risk.

**Table 1:** Performance metrics and 95% confidence intervals (CIs) of the gradient-boosted decision trees model (Extreme Gradient Boosting) with the top 68 features, the Juniper fall risk assessment score, and other machine learning models (logistic regression and multilayered perceptron) for the 3-month prediction of fall.

Variable	Extreme Gradient Boosting	Logistic regression	Multilayered perceptron	Juniper fall risk
Area under the receiver operating characteristic curve (95% CI)	0.846 (0.794-0.894)	0.711 (0.645-0.773)	0.697 (0.624-0.765)	0.621 (0.547-0.693)
Sensitivity (95% CI)	0.706 (0.577-0.833)	0.706 (0.553-0.859)	0.706 (0.571-0.833)	0.351 (0.217-0.485)
Specificity (95% CI)	0.848 (0.809-0.888)	0.614 (0.560-0.668)	0.612 (0.566-0.657)	0.883 (0.854-0.911)
Positive likelihood ratio	4.647	1.828	1.813	3.014
Negative likelihood ratio	0.346	0.479	0.481	0.733
Diagnostic odds ratio (95% CI)	13.400 (6.026-29.796)	3.816 (1.764-8.256)	3.766 (1.741-8.147)	4.113 (1.881-8.995)
True positive	24	24	24	12
True negative	268	194	193	279
False positive	48	122	123	37
False negative	10	10	10	22
F1^a^	0.393	0.262	0.248	0.289

^a^*F* score is defined as the harmonic mean between precision and recall.

### Reduction of Prediction Window

The ROC curves for the 2-month prediction window on the hold out test set are presented in Figures 3C and 3D. The XGBoosting model exhibited the highest performance with an AUROC of 0.753. The logistic regression and multilayered perceptron models demonstrated AUROC of 0.690 and 0.678, respectively. The AUROC associated with the Juniper fall risk assessment score was 0.582. Table S6 in Multimedia Appendix 1 presents additional performance metrics. Figure S1 in Multimedia Appendix 1 shows the XGBoosting SHAP plot for the 2-month prediction window.

### Separating Training and Testing Data Set by Facility Type

The EHR data set used in this study contained data from various care facilities. As part of the post hoc analyses and external validation, we separated the training and test sets based on facility type. The ROC curves for the models are shown in Figures 3E and 3F, showing that the XGBoosting AUROCs were higher than all other predictors for both the skilled nursing facility (0.716) and assisted living facility (0.740) test sets.

### Modifying Input Features

**Figure 5:**
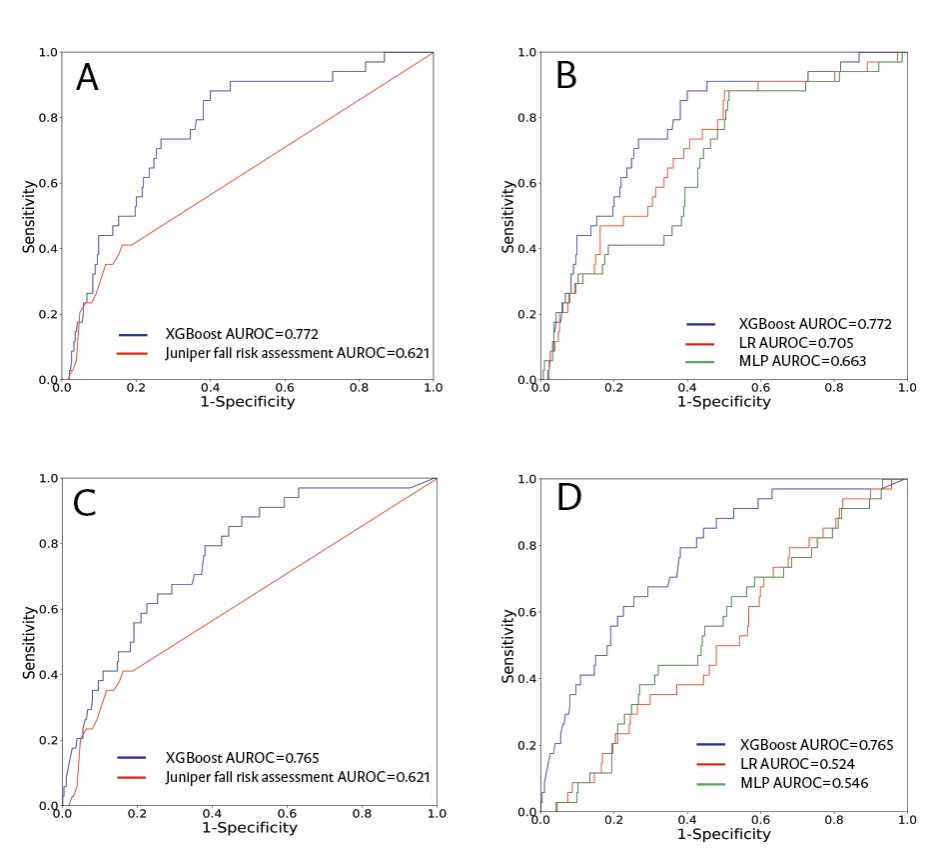
(A) Comparison between receiver operating characteristic (ROC) curves of Extreme Gradient Boosting (XGBoost) without vital signs and Juniper the fall risk model. (B) Comparison between ROC curves of XGBoost and other machine learning ML models without vital signs. (C) Comparison between ROC curves of XGBoost using only demographic information (age and sex) and vital signs and the Juniper fall risk model. (D) ROC curve of three ML models using demographic information and vital signs. AUROC: area under the receiver operating characteristic; ML: machine learning; MLP: multilayered perceptron; Logistic: Logistic Regression.

The ROC curves associated with the modified input features (3-month prediction window) are shown in Figure 5. When all variables related to vital signs were removed, the XGBoosting model maintained the highest performance with an AUROC of 0.772. Similarly, when using only demographic information (age and sex) and vital signs as input features, the XGBoosting model achieved the best performance, with an AUROC of 0.765. The SHAP plots of the models with the modified features are shown in Figures S2 and S3 in Multimedia Appendix 1.

## Discussion

### Summary of the Study

We developed an EHR-based ML model for short-term fall prediction in different long-term care facilities. Initially, 250 features were extracted from the EHR data, although only 68 features passed the initial selection process based on the feature importance metric. These features were used to train the final XGBoosting, logistic regression, and multilayered perceptron models. Using individual data collected from the residents’ EHR system, XGBoosting outperformed the Juniper fall risk assessment tool, which yielded only an AUROC of 0.621 versus 0.846 for XGBoosting. XGBoosting achieved a good trade-off in balancing the true positive and true negative rates (Table 1), outperforming the 2 other baseline ML models in both metrics.

### Best EHR-Based Features for Fall Prediction

Unlike standard fall risk assessment tools, MLA models can flag the importance of individual variables in predicting fall risk. The number of active medications was identified as the most significant feature associated with a higher fall incidence, followed by a resident’s number of active diseases and weight changes. The impact of the number of medications on fall incidents has been reported by previous observations in nursing homes, demonstrating that fall risk is associated with polypharmacy regimens that include at least one fall-increasing drug [41]. The 68 selected (out of 250) best features included several well-established fall risk factors, such as age, sex, history of falls, benzodiazepine, and antiepileptic medications. Except for the number of active medications, active diseases, hypertension, weight, and age, all other features with the highest ranking were measurements of vital signs, which were not used in the Juniper fall assessment. The most significant vital sign measure was mean diastolic blood pressure, with higher values inversely correlated with fall risk. Given that most of our study participants were women, this finding is also in line with a previous study on the relationship between blood pressure and falls in community-dwelling adults aged ≥60 years [50], where an increase in diastolic and systolic blood pressure reduced the risk of falls in women. The negative correlation between the number of active diseases and fall risk was likely because of the expected mobility restrictions of residents with multiple concurrent comorbidities. Separating weakness, dizziness, and unsteadiness due to other comorbidities did not affect the performance. Including the difference between consecutive vital sign measurements as individual features also did not improve the performance; therefore, we removed these features from the feature matrix to simplify our model.

### Reduction of Prediction Window

Although the performance of all 3 MLAs and the comparator risk stratification tool used by Juniper relapsed after reducing the prediction window to 2 months, the XGBoosting model continued to exhibit the highest performance. The observed performance decline associated with the 2-month prediction window was likely because of the lack of data, as illustrated in Figure 2B (loss of positive cases from 153 to 80). The optimal prediction window (3 months) was selected based on the frequency of the data present in the EHR. Owing to the importance of vital signs in predicting short-term fall risks, more frequent and consistent collection of these variables may allow shorter prediction windows without losing accuracy.

### External Validation

In addition to our primary model, which used EHR data from various facilities for training and testing, we explored other models in which one of the facilities was excluded from the training set but used for external validation. In both test cases, the XGBoosting-based model outperformed other ML algorithms. The XGBoosting AUROC in the assisted living facility test case was slightly higher (0.740) than that in the skilled nursing facility test case (0.716). This difference may be explained by the presence of a wider range of medication and comorbidities and more frequently measured vital signs in skilled nursing facility residents, making this cohort potentially a better training set for other facilities with fewer disabilities and medical conditions in their residents. In general, individuals living in skilled nursing facilities demand a higher level of nursing care and assistance with their daily activities than residents in assisted living communities or independent living facilities. In this study, the skilled nursing facility fall incidents were approximately 1.4 times higher than those of independent living facility fall incidents, which is consistent with previous epidemiological reports [28].

### Impact of Vital Signs

Several previous studies have identified history of falls as one of the most prominent risk factors for falls [51,52]. In our cohort, the history of falls was among the 68 preselected features, although it did not always rank among the top 20. When removing vital signs from the input features, fall history, lower extremity fractures, dizziness, and vertigo appeared among the top-ranking features. Moreover, our findings suggest that the combination of vital signs with traditional risk factors can achieve higher prediction accuracy than using either group of features alone.

### Implications of Findings

The Centers for Medicare and Medicaid Services facilities are required to complete a fall risk assessment upon residents’ admission, using the minimum data set (MDS) tool. Given that reassessments are not conducted frequently [53-55], changes in a resident’s fall risk status may not be detected in a timely manner. The United States Centers for Disease Control has established the Stopping Elderly Accidents, Deaths & Injuries program [56] to evaluate clinical fall risk prevention programs and provide best practice recommendations. The existence of the Stopping Elderly Accidents, Deaths & Injuries program highlights the gap in existing risk prediction and risk stratification tools that are generalizable and have high accuracy. Commonly used fall risk stratification tools, such as the Morse Fall Scale, St Thomas’s risk assessment tool in falling inpatients, and the Berg Balance Scale [31,57], rely on a clinician’s assessment of gait, mental status, and mobility. As Juniper facilities have their own internal fall risk assessment, this was an appropriate comparator for our study, as opposed to comparing our MLA with any of the aforementioned tools. Traditional models overlook other significant fall risk factors that we identified in our ML models, such as diastolic blood pressure and respiratory rate, which are measurements easily obtained from EHR data without interrupting the clinical workflow. The sensitivity of these tools is inconsistent across the literature, ranging from 33.33% to 95% [58]. Using the MDS data set, the study by Marier et al [53] examined the use of MDS in tandem with EHR data, as the latter incorporates more frequent clinical measurements that may indicate changes in an individual’s health status, thus potentially providing improved risk assessment [51]. The study determined that the use of EHR data improved fall risk identification by 13% compared with using only MDS data, which may be attributed to the fact that EHR is updated more frequently. Long-term care facilities have a lower rate of EHR implementation and use than other clinical settings (18%-48%). Using an XGBoosting-ML approach with EHR data without vital signs, the study by Ye et al [27] predicted fall incidents in hospitalized patients >65 years of age. At the 1- and 2-month prediction windows, they were able to predict only 55% to 58% of falls, which may be attributed to the lack of vital signs in their model. The EHR-based ML models for fall prediction are also cost-effective. Early identification of high-risk individuals can enable prompt intervention, such as the removal of environmental hazards or providing additional assistance with specific daily activities (bathroom visits), behavioral therapy, and exercise for muscle strengthening.

### Study Limitations and Future Directions

Our study has several limitations. First, this study was restricted to retrospective data with highly imbalanced classes, missing data, and a higher prevalence of women in the data set. Although the ML algorithms implemented several optimization parameters to overcome these shortcomings (see Table S6 in Multimedia Appendix 1 for model performance evaluation in women and men), the impact of EHR data quality and class balance on model performance could not be evaluated in this study. Regarding medication use, previous research has identified a dose-response relationship between medications, particularly benzodiazepines [59], and fall risk. Given that our data did not provide structured information about medication dosage, we were not able to include dosage as an input feature. The lack of standardized data collection methods for residents of different types of communities poses another methodological challenge. In particular, the collection of vital sign measures was highly variable across facilities and individuals. Given the importance of vital signs for fall risk, a more frequent and consistent collection of vital signs could leverage the extraction of fine-grained features (change in diastolic pressure between measurements). Although the study findings were validated across different types of Juniper care facilities, the generalizability of the findings outside Juniper Communities warrants further investigation. More than half of the individuals’ fall incidences were not recorded using ICD codes (gold standard), and a manual search of their progress notes was required to identify these falls. Other study limitations include the lack of information regarding the severity of medical conditions and the potential that some fall events were missing from the EHRs. Further research and the use of our MLAs for fall risk prediction before implementation are warranted. Future directions for this research will focus on developing and implementing more interpretable ML models, such as the explainable boosting machine or deep learning techniques (eg, recurrent neural networks). This will allow for the incorporation of additional forms of digitized physiological and behavioral data that may be relevant to fall risks. Recurrent neural networks can process sequences of input data with variable lengths, making them applicable for recognizing patterns in electrocardiogram signals, motion, and speech notes [60-63].

### Conclusions

This study shows that the XGBoosting technique can use a large number of features from EHR data to make short-term fall predictions with a better performance than conventional fall risk assessments and other ML models. The integration of routinely collected EHR data, particularly vital signs, into fall prediction models may generate a more accurate fall risk surveillance than models without vital signs. Our data support the use of ML models for dynamic, cost-effective, and automated fall prediction in different types of senior care facilities.

## Appendix

Multimedia Appendix 1 Supplementary materials.

## Abbreviations

AUROC: area under the receiver operating characteristic
EHR: electronic health record
ICD: International Classification of Diseases
MDS: minimum data set
ML: machine learning
MLA: machine learning algorithm
ROC: receiver operating characteristic
SHAP: Shapely Additive Explanations
XGBoosting: Extreme Gradient Boosting
