# Supplementary material for: Predicting Falls in Long-term Care Facilities: Machine Learning Study
Source: JMIR Aging. 2022 Apr 1;5(2):e35373. doi: 10.2196/35373 (PMC9015781; doi:10.2196/35373)

**Multimedia Appendix I**

**(Supplementary Tables and Figures)**

*Supplementary Table 1.* **Machine learning** input features. Bolded features are those that were preselected based on results from the feature importance analysis.

| Feature Group | Feature | Feature group | Feature |
| --- | --- | --- | --- |
| **Vitals** | **Respiratory rate [beats per minute]**  **Diastolic blood pressure [mmHg]**  **Systolic blood pressure [mmHg]**  **Heart rate [beats per minute]**  **Temperature [degree C]** | **Labs** | Glucose  CO2  Sodium  Creatinine  Potassium  Calcium  Chloride  Blood urea nitrogen  Albumin  Cholesterol |
| **Physical** | **Height [in]**  **Weight [lbs]**  **Lower extremity fracture or dislocation**  **History of fall** | **Demographics** | **Age**  **Sex** |
| **Comorbidities** | Chronic kidney disease  Diabetes  Cancer  Hypertension  **Chronic heart failure**  Dementia  Chronic obstructive pulmonary disease  Myocardial infarction  Arrhythmia  Other mental behaviors or disorders  Schizophrenia or psychosis  Mood or affective disorders or somatoform  Movement disorder  Lower extremity fracture  Vertebrae and neck fracture  Upper extremity fracture  Healed fractures  **Stroke and cerebrovascular**  History of fall  Abnormal gait  Weakness, dizziness, and unsteadiness  vertigo | **Medications** | Antiepileptic, anticonvulsant  **Benzodiazepine**  Antidepressants  Narcotics  Diuretics  Beta blockers  Anticholinergics antimuscarinics antispasmodics  Antipsychotics  Neuromuscular blocking agents  Antihistamines  Calcium channels  Antiarrhythmics  **Angiotensin converting enzyme inhibitors**  Alpha adrenergic blocking agents  Sedative hypnotic  **Number of active medications** |
| **Assessments** | AIMS - Abnormal Involuntary Movement Scale, Brief Interview for Mental Status (3.0 BIMS), Cornell Scale for Depression in Dementia, Hamilton Scale for Depression (17 point), Pain Evaluation in Advanced Dementia | | |

*Supplementary Table 2* (7 pages)

| Disease | ICD-10 | ICD-9 |
| --- | --- | --- |
| Dementia | F01 Vascular dementia  F02 Dementia in other diseases classified elsewhere  F03 Unspecified dementia  F04 Amnestic disorder due to known physiological condition  G30 Alzheimer disease  F00.0 Dementia in Alzheimer disease  G31 Frontotemporal dementia  G32 Other degenerative disorders of nervous system in diseases classified elsewhere | 290 Dementias  331.0 Alzheimer disease  331.1 Frontotemporal dementia  331.2 Senile degeneration of brain  331.7 Cerebral degeneration in diseases classified elsewhere  331.8 Other cerebral degeneration  331.9 Cerebral degeneration, unspecified  331.82 Dementia with Lewy bodies  294.1 Dementia in conditions classified elsewhere  294.2 Dementia, unspecified  292.82 Drug-induced persisting dementia  291.2 Alcohol-induced persisting dementia  "348.3 Encephalopathy, unspecified " |
| References | https://apps.who.int/iris/handle/10665/37958  https://www.sciencedirect.com/science/article/abs/pii/S0895435604001647  https://www.sciencedirect.com/science/article/abs/pii/S0967586814000435  https://www.hindawi.com/journals/jce/2010/569517/  https://content.iospress.com/articles/journal-of-alzheimers-disease/jad170518 | |

| Schizophrenia / psychosis / delusional | F20 Schizophrenia  F21 Schizotypal disorder  F22 Persistent delusional disorders  F23 Acute psychotic disorder  F24 Induced delusional disorder  F25 Schizoaffective disorders  F28 Other psychotic disorder not due to a substance or known physiological condition  F29 Unspecified psychosis not due to a substance or known physiological condition | 295 Schizophrenic disorders  301.22 Schizotypal personality disorder  297.0 Paranoid state, simple  297.1 Delusional disorder  297.2 Paraphrenia  298.3 Acute paranoid reaction  298.4 Psychogenic paranoid psychosis  298.8 Other and unspecified reactive psychosis  297.3 Shared psychotic disorder  295.7 Schizoaffective disorder, unspecified  298.9 Unspecified psychosis |
| --- | --- | --- |
| Reference | https://apps.who.int/iris/handle/10665/37958 | |

| Disease | ICD-10 | ICD-9 |
| --- | --- | --- |
| Mood / affective disorders /somatoform | F30 Manic episode  F31 Bipolar disorder  F32 Major depressive disorder, single episode  F33 Major depressive disorder, recurrent  F34 Persistent mood [affective] disorders  F39 Unspecified mood [affective] disorder  F40 Phobic anxiety disorders  F41 Other anxiety disorders  F42 Obsessive-compulsive disorder  F43 Reaction to severe stress, and adjustment disorders  F38 Other Persistent mood [affective disorder]  F44 Dissociative and conversion disorders  F45 Somatoform disorders | 296 Episodic mood disorders  300 Anxiety, dissociative, and somatoform disorders |
| Reference | https://apps.who.int/iris/handle/10665/37958 | |

| Movement disorders and neurological disease with movement disorder | G10 Huntington's disease  G11 Hereditary ataxia  G12 Spinal muscular atrophy and related syndromes  G13 Systemic atrophies primarily affecting central nervous system in diseases classified elsewhere  G14 Postpolio syndrome  G20 Parkinson disease | 333.4 Huntington's chorea  334 Spinocerebellar diseases  335.1 Spinal muscular atrophy  138 Late effects of acute poliomyelitis  332 Parkinson disease |
| --- | --- | --- |
| Reference | https://apps.who.int/iris/handle/10665/37958 | |

| Disease | ICD-10 | ICD-9 |
| --- | --- | --- |
| Other or Unspecified mental/  behavioral Disorders | F05 Delirium due to known physiological condition  F06 Other mental disorders due to known physiological condition  F07 Personality and behavioral disorders due to known physiological condition  F09 Unspecified mental disorder due to known physiological condition  F48 Other nonpsychotic mental disorders  F54 Psychological and behavioral factors associated with disorders or diseases classified elsewhere  F59 Unspecified behavioral syndromes associated with physiological disturbances and physical factors  F99 Mental disorder, not otherwise specified  F48 Other nonpsychotic mental disorders  F51 Sleep disorders not due to a substance or known physiological condition | 293.0 Delirium due to conditions classified elsewhere  293.1 Subacute delirium  293.8 Other specified transient mental disorders due to conditions classified elsewhere  293. 9 Unspecified transient mental disorder in conditions classified elsewhere  294.8 Persistent mental disorders due to conditions classified elsewhere  294.9 Unspecified persistent mental disorders due to conditions classified elsewhere  301.8 Other personality disorders  301.9 Unspecified personality disorders  310 Specific nonpsychotic mental disorders due to brain damage  316 Psychic factors associated with diseases classified elsewhere  306.8 Other specified psychophysiological malfunction  293.9 Unspecified transient mental disorder in conditions classified elsewhere  780.09 Other alteration of consciousness |
| Reference | https://apps.who.int/iris/handle/10665/37958 | |

| Gait | R26.9 Unspecified abnormalities of gait and mobility  R26 Abnormalities of gait and mobility  R26.89 Other abnormalities of gait and mobility  R26.0 Ataxic gait  R26.8 Other abnormalities of gait and mobility  R27 Other lack of coordination | 781.2 Abnormality of gait  781.3 lack of coordination |
| --- | --- | --- |
| Reference | https://movementdisorders.onlinelibrary.wiley.com/doi/full/10.1002/mdc3.12474  https://www.proquest.com/openview/fabc59e9a0cb49e28e3493736a6afd98/1?pq-origsite=gscholar&cbl=40575  https://digitalcommons.csbsju.edu/nursing_students/3/ | |

| Disease | ICD-10 | ICD-9 |
| --- | --- | --- |
| Vertebra and neck fracture | M80.08 Age-related osteoporosis with current pathological fracture, vertebra(e)  S12 Fracture of cervical vertebra and other parts of neck  S22 Fracture of rib(s), sternum and thoracic spine  S02 Fracture of skull and facial bones | 733.13 Pathologic fracture of vertebrae  800 Fracture of vault of skull  801 Fracture of base of skull  802 Fracture of face bones  803 Other and unqualified skull fractures  804 Multiple fractures involving skull or face with other bones  805 Fracture of vertebral column without mention of spinal cord injury  806 Fracture of vertebral column with spinal cord injury  807 Fracture of rib(s) sternum larynx and trachea  809 Ill-defined fracture of trunk |
| Reference | https://link.springer.com/content/pdf/10.1007/s00198-015-3277-9.pdf  https://link.springer.com/article/10.1007/s00198-004-1696-0  https://onlinelibrary.wiley.com/doi/abs/10.1197/j.aem.2005.07.038  https://www.nature.com/articles/3101003  https://ard.bmj.com/content/65/3/335.short | |

| Healed fractures | Z87.81 Personal history of (healed) traumatic fracture  Z87.312 Personal history of (healed) stress fracture  Z87.310 Personal history of (healed) osteoporosis fracture  Z87.311 Personal history of (healed) pathological NEC fracture | v15.51 Personal history of traumatic fracture  v13.52 Personal history of stress fracture  v13.51 Personal history of pathologic fracture |
| --- | --- | --- |
| Reference | https://web.archive.org/web/20180411124524id_/http://www.nachimsonadvisors.com/Documents/ICD-10%20Impacts%20on%20Providers.pdf  https://link.springer.com/article/10.1007/s00198-013-2369-7  https://link.springer.com/article/10.1007/s00198-016-3655-y  https://www.apma.org/files/FileDownloads/Webinar%205b%20-%20Freedman.pdf | |

| Disease | ICD-10 | ICD-9 |
| --- | --- | --- |
| Lower extremity and hip fracture | M80.05 Age-related osteoporosis with current pathological fracture, femur  M84.45 Pathological fracture, femur and pelvis  M84.65 Pathological fracture in other disease, pelvis and femur  M84.75 Atypical femoral fracture  M80.06 Age-related osteoporosis with current pathological fracture, lower leg  M84.46 Pathological fracture, tibia and fibula  M84.66 Pathological fracture in other disease, tibia and fibula  S72 Fracture of femur  S82 Fracture of lower leg, including ankle  S92 Fracture of foot and toe  S32 Fracture of lumbar spine and pelvis  M24.35 Pathological dislocation of hip, not elsewhere classified  M24.36 Pathological dislocation of knee, not elsewhere classified  M24.37 Pathological dislocation of ankle and foot, not elsewhere classified | 733.14 Pathologic fracture of neck of femur  733.15 Pathologic fracture of other specified part of femur  733.16 Pathologic fracture of tibia or fibula  v54.23 Aftercare for healing pathologic fracture of hip  v54.24 Aftercare for healing pathologic fracture of leg, unspecified  v54.25 Aftercare for healing pathologic fracture of upper leg  v54.26 Aftercare for healing pathologic fracture of lower leg  733.97 Stress fracture of femur  820 Fractures of neck of femur  821 Fracture of other and unspecified parts of femur  823 Fracture of tibia and fibula  733.97 Stress fracture of shaft of femur  733.98 Stress fracture of pelvis  733.93 Stress fracture of tibia or fibula  733.96 Stress fracture of femoral neck  733.94 Stress fracture of the metatarsals  824 Fracture of ankle  825 Fracture of one or more tarsal and metatarsal bones  826 Fracture of one or more phalanges of foot  827 Other, multiple and ill-defined fractures of lower limb  820 Fracture of neck of femur  822 Fracture of patella  821 Fracture of other and unspecified parts of femur  808 fracture pelvis  828 Multiple fractures involving both lower limbs lower with upper limb and lower limb(s) with rib(s) and sternum  718.25 Pathological dislocation of joint, pelvic region and thigh  718.26 Pathological dislocation of joint, lower leg  718.27 Pathological dislocation of joint, ankle and foot |

| Disease | ICD-10 | ICD-9 |
| --- | --- | --- |
| Cancer | Z85  Z17  C00-C24  C30-c34  C37-C41  C43-C58  C60-C80  C7A  C7b  C81-C86  C88  C90-96 | 140-165  170-176  180-209 |
| Reference | https://www.sciencedirect.com/science/article/pii/S2405852116300179  https://www.sciencedirect.com/science/article/pii/S092375341941137X  https://www.hindawi.com/journals/jce/2010/569517/ | |

| Stroke and cerebrovascular | I60 Nontraumatic subarachnoid hemorrhage  I61 Nontraumatic intracerebral hemorrhage  I62 Other and unspecified nontraumatic intracranial hemorrhage  I63 Cerebral infarction  I65 Occlusion and stenosis of precerebral arteries, not resulting in cerebral infarction  I66 Occlusion and stenosis of cerebral arteries, not resulting in cerebral infarction  I67 Other cerebrovascular diseases  I68 Cerebrovascular disorders in diseases classified elsewhere  I69 Sequelae of cerebrovascular disease  H34.1 Central retinal artery occlusion  H34.2 Other retinal artery occlusions  197.81 Intraoperative cerebrovascular infarction  197.82 Postprocedural cerebrovascular infarction | 430 Subarachnoid hemorrhage  431 Intracerebral hemorrhage  432 Other and unspecified intracranial hemorrhage  434 Occlusion of cerebral arteries  433 Occlusion and stenosis of precerebral arteries  435 Transient cerebral ischemia  437 Other, ill-defined cerebrovascular diseases  436 Acute, but ill-defined, cerebrovascular disease  438 Late effect of cerebrovascular disease  362.31 Central retinal artery occlusion convert  362.32 Retinal arterial branch occlusion  362.33 Partial retinal arterial occlusion |
| --- | --- | --- |
| Ref | https://www.ahajournals.org/doi/10.1161/STR.0000000000000366  https://link.springer.com/article/10.1007/s10072-006-0721-9  https://journals.plos.org/plosone/article?id=10.1371/journal.pone.0135834  https://www.ahajournals.org/doi/full/10.1161/01.STR.0000174293.17959.a1  https://onlinelibrary.wiley.com/doi/full/10.1111/aos.14369 | |

| Disease | ICD-10 | ICD-9 |
| --- | --- | --- |
| Upper extremity fracture | M80.03 Age-related osteoporosis with current pathological fracture, forearm  M84.43 Pathological fracture, ulna and radius  M84.63 Pathological fracture in other disease, ulna and radius  M80.01 Age-related osteoporosis with current pathological fracture, shoulder  M80.02 Age-related osteoporosis with current pathological fracture, humerus  M84.41 Pathological fracture, shoulder  M84.42 Pathological fracture, humerus  M84.61 Pathological fracture in other disease, shoulder  M84.62 Pathological fracture in other disease, humerus  S42 Fracture of shoulder and upper arm  S52 Fracture of forearm  S62 Fracture at wrist and hand level  M24.31 Pathological dislocation of shoulder, not elsewhere classified  M24.32 Pathological dislocation of elbow, not elsewhere classified  M24.33 Pathological dislocation of wrist, not elsewhere classified  M24.34 Pathological dislocation of hand, not elsewhere classified | 733.12 Pathologic fracture of distal radius and ulna  733.11 Pathologic fracture of humerus  718. 21 Pathological dislocation of joint, shoulder region  718.22 Pathological dislocation of joint, upper arm  718.23 Pathological dislocation of joint, forearm  718.24 Pathological dislocation of joint, hand  810 Fracture of Clavicle  811 Fracture of Scapula  812 Fracture of Humerus  813 Fracture of radius and ulna  814 Fracture of Carpal bone(s)  815 Fracture of metacarpal bone(s)  816 Fracture of one or more Phalanges of the hand  817 Multiple Fracture of hand bones  818 Ill-defined fractures of upper limb  819 Multiple fractures involving both upper limbs, and upper limbs with rib and sternum  v54.20 Aftercare for healing pathologic fracture of arm, unspecified  v54.21 Aftercare for healing pathologic fracture of upper arm  v54.22 Aftercare for healing pathologic fracture of lower arm |
| reference | https://link.springer.com/content/pdf/10.1007/s00198-015-3277-9.pdf  https://link.springer.com/article/10.1007/s00198-004-1696-0  https://link.springer.com/article/10.1007/s00198-004-1686-2  https://europepmc.org/article/med/10666853  https://onlinelibrary.wiley.com/doi/abs/10.1111/ijcp.14549  https://www.sciencedirect.com/science/article/abs/pii/S109185311730068X  https://getd.libs.uga.edu/pdfs/extavour_rian-marie_201505_phd.pdf | |

*Supplementary Table 3.* Software Development Environment for ML algorithms

| **Package/Libraries** | **Version** |
| --- | --- |
| Python | 3.6.10 |
| Pandas | 1.1.5 |
| Numpy | 1.19.5 |
| Scikit-learn | 0.24.2 |
| XGBoost | 1.2.0 |

*Supplementary Table 4*. Fall prevalence in different types of long-term care facilities. Description of the facilities: individuals who live in Skilled Nursing Facilities demand a higher level of nursing care and assistance with their daily activities than residents in Assisted Living Facilities who are no longer able to reside safely at home but don’t require the same level of clinical care. Residents in Independent Living Facilities can live independently without receiving care from nursing staff.

| **Facility Type** | **Fall Residents (%)** | **non-Fall Residents (%)** |
| --- | --- | --- |
| Skilled nursing facility | 49 (10.02) | 440 (89.98) |
| Assisted living facility | 45 (8.54) | 482 (91.46) |
| Independent living facility | 5 (7.24) | 64 (92.75) |
| Others | 54 (8.13) | 610 (91.87) |

*Supplementary Table 5.* Combined data used to train and test models

| **Variables** | **Non-Fall (%), N = 1596** | **Fall (%), N = 153** | ***P*-value** |
| --- | --- | --- | --- |
|  | | | |
| **Age** |  |  |  |
| 60-70 | 154 (9.6) | 6 (3.9) | 0.03 |
| 70-80 | 231 (14.5) | 27 (17.6) | 0.35 |
| 80-90 | 581 (36.4) | 55 (35.9) | 0.99 |
| 90-100 | 606 (38.0) | 64 (41.8) | 0.53 |
| 100+ | 24 (1.5) | 1 (0.7) | 0.72 |
| **Sex** |  |  |  |
| Female | 1,039 (65.1) | 101 (66.0) | 0.95 |
| Male | 544 (34.1) | 50 (32.7) | 0.87 |
| Unknown | 13 (0.8) | 2 (1.3) | 0.39 |
| **Comorbidities** |  |  |  |
| Chronic Kidney Disease | 261 (16.4) | 30 (19.6) | 0.38 |
| Renal Failure | 61 (3.8) | 4 (2.6) | 0.65 |
| Diabetes | 377 (23.6) | 25 (16.3) | 0.11 |
| Malignant Cancer | 255 (14.1) | 25 (16.3) | 0.55 |
| Hypertension | 1,121 (70.2) | 96 (62.7) | 0.42 |
| Chronic Heart Failure | 264 (16.5) | 30 (19.6) | 0.44 |
| cognitive impairment | 806 (50.5) | 78 (51.0) | 0.94 |
| Pulmonary Disease | 208 (13.0) | 16 (10.5) | 0.53 |
| Myocardial Infarction | 49 (3.1) | 3 (2.0) | 0.62 |
| Arrhythmia | 432 (27.1) | 37 (24.2) | 0.64 |
| Schizophrenia or Psychosis | 126 (7.9) | 7 (4.6) | 0.20 |
| Mood/affective disorders | 789 (49.4) | 73 (47.7) | 0.88 |
| Movement Disorders | 99 (6.2) | 5 (3.3) | 0.21 |
| Lower Extremity Fractures | 135 (8.5) | 25 (16.3) | 0.007 |
| Vertebra and Neck Fracture | 50 (3.1) | 5 (3.3) | 0.81 |
| Upper Extremity Fracture | 57 (3.6) | 2 (1.3) | 0.23 |
| Stroke and Cerebrovascular | 255 (16.0) | 17 (11.1) | 0.20 |
| Abnormal Gait | 324 (20.3) | 31 (20.3) | 0.99 |
| Weakness/Dizziness | 331 (20.7) | 32 (20.9) | 0.99 |
| Vertigo | 27 (1.7) | 5 (3.3) | 0.20 |

*Supplementary Table 6.* Performance metrics and 95% confidence interval of the XGBoost with top 68 features, Juniper fall risk assessment score, and baseline ML models logistic regression and multilayered perceptron for the *two-month* prediction window. Abbreviations used: LR: Likelihood Ratios; DOR: Diagnostic Odds Ratio; TP: True Positive; TN: True Negative; FP: False Positive; FN: False Negative. F1: F1-score, defined as harmonic mean between precision and recall

|  | **XGBoost** | **Logistic regression** | **Multilayered Perceptron** | **Juniper Fall Risk** |
| --- | --- | --- | --- | --- |
| **AUROC** | 0.753  (0.637 - 0.865) | 0.690  (0.566 - 0.791) | 0.678  (0.549 - 0.787) | 0.582  (0.469 - 0.700) |
| **Sensitivity** | 0.706  (0.500 - 0.889) | 0.706  (0.500 - 0.882) | 0.706  (0.500 - 0.875) | 0.353  (0.154 - 0.556) |
| **Specificity** | 0.688  (0.648 - 0.733) | 0.730  (0.687 - 0.77) | 0.700  (0.655 - 0.740) | 0.826  0.790 - 0.861) |
| **LR+** | 2.260 | 2.611 | 2.350 | 2.026 |
| **LR-** | 0.427 | 0.403 | 0.420 | 0.783 |
| **DOR** | 5.284 | 6.480 | 0.420 | 2.586 |
| **TP** | 12 | 12 | 12 | 6 |
| **TN** | 229 | 243 | 233 | 275 |
| **FP** | 104 | 90 | 100 | 58 |
| **FN** | 5 | 5 | 5 | 11 |
| **F1** | 0.180 | 0.201 | 0.186 | 0.148 |

Supplementary Table 7: Model performance in test sets separated by sex. Abbreviations used: LR: Likelihood Ratios; DOR: Diagnostic Odds Ratio; TP: True Positive; TN: True Negative; FP: False Positive; FN: False Negative. F1: F1-score, defined as harmonic mean between precision and recall

|  | XGBoost (Men) | XGBoost (Women) |
| --- | --- | --- |
| AUROC | 0.830  (0.708 - 0.938) | 0.845  (0.788 - 0.902) |
| Sensitivity | 0.75  (0.505 - 0.995) | 0.727  (0.541 - 0.913) |
| Specificity | 0.824  (0.75 - 0.898) | 0.769  (0.712 - 0.826) |
| TP | 9 | 16 |
| TN | 84 | 163 |
| FP | 18 | 49 |
| FN | 3 | 6 |
| F1 | 0.462 | 0.368 |

*Supplementary Figure 1.* Extreme Gradient Boosting (XGBoost) SHAP plot for two-month prediction window. Model input variables are ranked in descending order of feature importance. The y-axis on the SHAP plot presents the features in order of importance from top to bottom. The SHAP values on the x-axis quantify the magnitude and direction in which each feature impacts the model prediction. SHAP: Shapely Additive Explanations; max: maximum; min: minimum; SD: standard deviation.


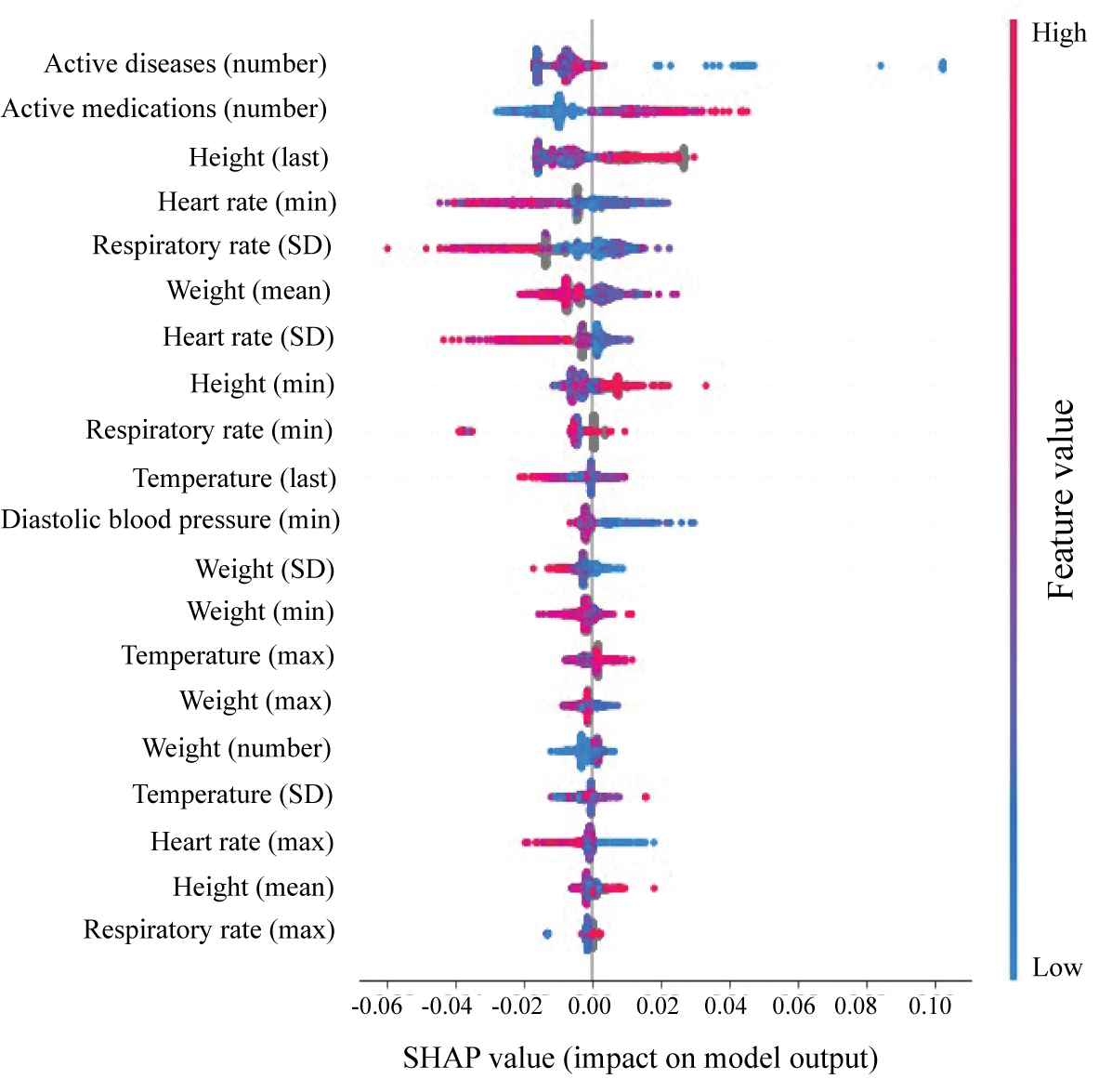


*Supplementary Figure 2 (modified input features)*: SHAP plot for XGBoostmodel (three-months prediction) without vital signs as input feature. The y-axis on the SHAP plot presents the features in order of importance from top to bottom. The SHAP values on the x-axis quantify the magnitude and direction in which each feature impacts the model prediction. SHAP: Shapely Additive Explanations.


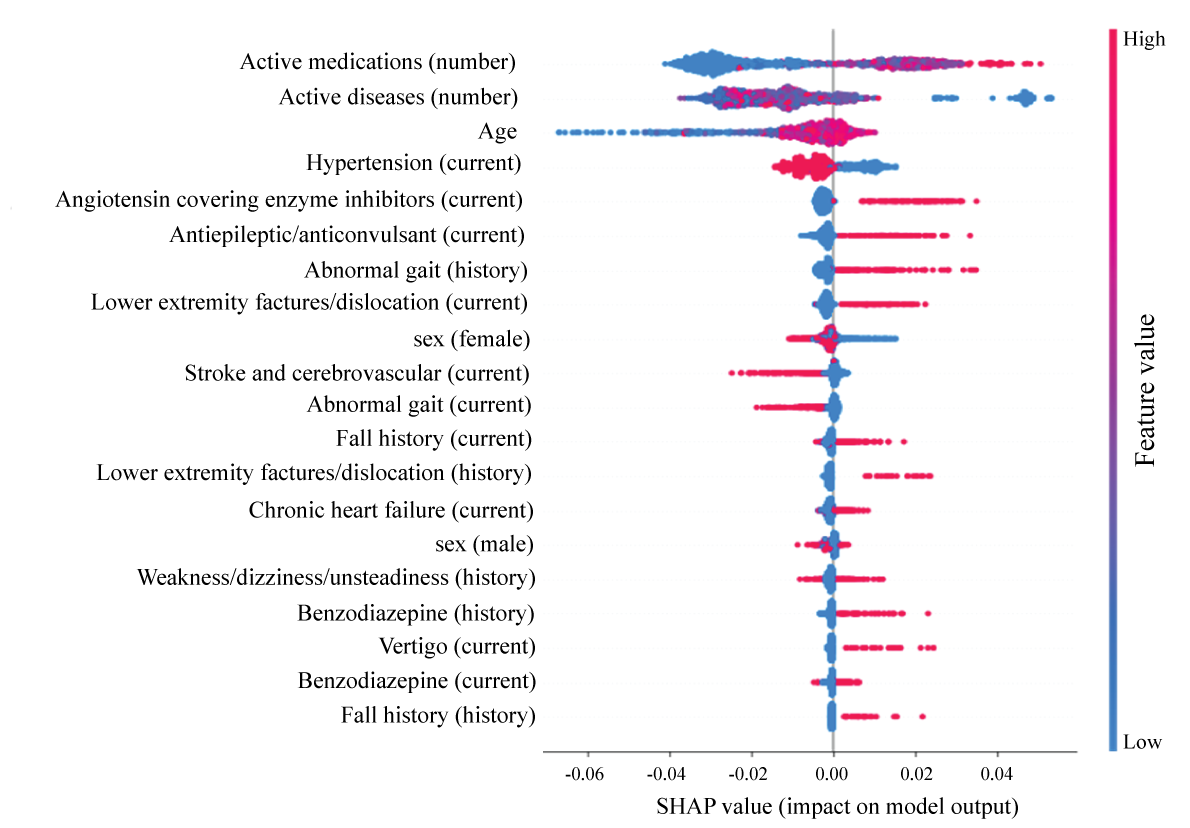


*Supplementary Figure 3 (modified input features):* SHAP plot for XGBoost model (three-month prediction) using only demographic information (age, sex) and vital signs as input features. The y-axis on the SHAP plot presents the features in order of importance from top to bottom. The SHAP values on the x-axis quantify the magnitude and direction in which each feature impacts the model prediction. SHAP: Shapely Additive Explanations; max: maximum; min: minimum; SD: standard deviation.


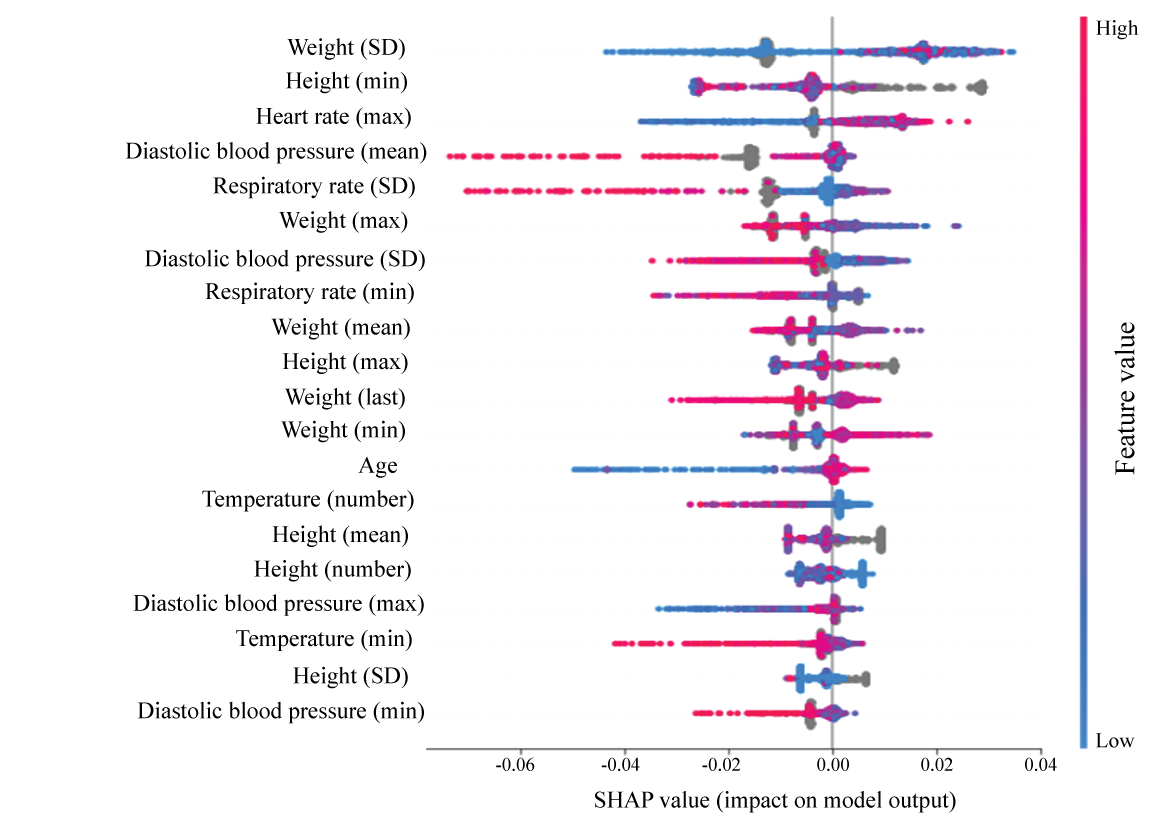

Supplement: Multimedia Appendix 1 [file aging_v5i2e35373_app1.docx]
